# Everyday Stressors, Discrimination, and Health Among LGBTQ+ Older Adult Caregivers of Adults Living with MCI/ADRD

**DOI:** 10.1093/geroni/igaf122.3759

**Published:** 2025-12-31

**Authors:** Nik Lampe, Victoria Money, Kirsty Clark, Tara Mckay

**Affiliations:** University of South Florida, Tampa, Florida, United States; University of South Carolina, Columbia, South Carolina, United States; Vanderbilt University, Nashville, Tennessee, United States; Vanderbilt University, Nashville, Tennessee, United States

## Abstract

Using LGBTQ+ Social Networks, Aging, and Policy Study Wave 3 data (N = 982), this study examines how caregiving for individuals with mild cognitive impairment, Alzheimer’s disease, and related dementias (MCI/ADRD) impacts everyday stressors, discrimination, and health outcomes among LGBTQ+ older adults in the Southern United States. Using chi-square tests, Fisher’s exact tests, logistic regression, and Poisson regression models, we assessed associations of caregiver role with stress and health outcomes. We then conducted moderation analyses to test interactions between caregiver role and LGBTQ+ minority stress. LGBTQ+ caregivers of individuals with MCI/ADRD were three times more likely to experience proximal minority stress (p < 0.05), twice as likely to report suicidal ideation (p < 0.1), and more likely to report cognitive problems (p < 0.05) and high blood pressure (p < 0.05) than non-caregivers. Interaction effects suggest that minority stress may exacerbate mental health problems among MCI/ADRD caregivers. Findings underscore the need for targeted supports and interventions for LGBTQ+ MCI/ADRD caregivers.

